# Characterization of Three Different Unusual S-Layer Proteins from *Viridibacillus arvi* JG-B58 That Exhibits Two Super-Imposed S-Layer Proteins

**DOI:** 10.1371/journal.pone.0156785

**Published:** 2016-06-10

**Authors:** Matthias Suhr, Franziska L. Lederer, Tobias J. Günther, Johannes Raff, Katrin Pollmann

**Affiliations:** 1 Helmholtz-Zentrum Dresden-Rossendorf, Helmholtz Institute Freiberg for Resource Technology, Freiberg, Germany; 2 Helmholtz-Zentrum Dresden-Rossendorf, Institute of Resource Ecology, Dresden, Germany; ContraFect Corporation, UNITED STATES

## Abstract

Genomic analyses of *Viridibacillus arvi* JG-B58 that was previously isolated from heavy metal contaminated environment identified three different putative surface layer (S-layer) protein genes namely *slp*1, *slp*2, and *slp*3. All three genes are expressed during cultivation. At least two of the *V*. *arvi* JG-B58 S-layer proteins were visualized on the surface of living cells via atomic force microscopy (AFM). These S-layer proteins form a double layer with *p4* symmetry. The S-layer proteins were isolated from the cells using two different methods. Purified S-layer proteins were recrystallized on SiO_2_ substrates in order to study the structure of the arrays and self-assembling properties. The primary structure of all examined S-layer proteins lack some features that are typical for *Bacillus* or *Lysinibacillus* S-layers. For example, they possess no SLH domains that are usually responsible for the anchoring of the proteins to the cell wall. Further, the pI values are relatively high ranging from 7.84 to 9.25 for the matured proteins. Such features are typical for S-layer proteins of *Lactobacillus* species although sequence comparisons indicate a close relationship to S-layer proteins of *Lysinibacillus* and *Bacillus* strains. In comparison to the numerous descriptions of S-layers, there are only a few studies reporting the concomitant existence of two different S-layer proteins on cell surfaces. Together with the genomic data, this is the first description of a novel type of S-layer proteins showing features of *Lactobacillus* as well as of *Bacillus*-type S-layer proteins and the first study of the cell envelope of *Viridibacillus arvi*.

## Introduction

Surface layers (S-layer) are proteinaceous cell surface structures that are ubiquitously found at the cell surface of many bacteria and archaea. They form the outermost layer of the cell as a barrier and are largely determining their interaction with the environment including interaction with other organisms, exchange of nutrients and metabolic waste products, and the interaction with external stressors *e*. *g*. heavy metals [1]. In most cases S-layers are composed of identical (glyco)protein subunits with a molecular weight ranging from 40–200 kDa that form two-dimensional highly regular arrays of different symmetries on cells and once isolated in suspension or on suitable surfaces or interfaces [2,3]. S-layer protein subunits can be arranged in lattices with oblique (*p1*, *p2*), square (*p4*) or hexagonal (*p3*, *p6*) symmetry and lattices exhibit center-to-center spacings of 2.5 to 35 nm [2,4]. S-layer proteins are expressed on a high level. In some strains the presence of several S-layer genes within one genome has been described. Some of them are silent genes, some genes are sequentially expressed during growth, influenced by environmental conditions, and antigenic variation based on S-layer gene expression has been monitored [4,5,6,7,8,9,10]. In some cases cell envelopes that are composed of two different S-layer proteins have been described [11,12,13,14,15].

In case of *Bacillus anthracis*, S-layer expression changes during growth and different S-layer proteins are incorporated into the S-layer at different growth stages, exhibiting different symmetries [10,16]. In strains of *Aquaspirillum serpens*, *Brevibacillus brevis* as well as *Clostridium difficile* the presence of two superimposed S-layer lattices was reported [17,18,19,20]. In all these cases the existence of the double S-layer proteins was proven with thin-section preparations, freeze-etch preparations, and/or negative-staining of cells and cell wall fragments. There are numerous studies describing S-layer proteins of different bacterial strains and giving detailed genetic data. Despite the high diversity of their primary structure there are some features that are considered as typical for bacterial S-layer proteins. The subunit proteins of most bacterial S-layers are composed of weakly acidic amino acids (aa), contain 40–60% hydrophobic amino acids, and possess few or no sulfur-containing amino acids [3]. In most cases, S-layer proteins have isoelectric points (pI) in the weakly acid range. In contrast, S-layer proteins from Lactobacilli consist of many basic aa and possess pI-values ranging from 9.35–10.4 [21]. In comparison with other S-layer proteins, *Lactobacillus* S-layer proteins are also much smaller with molecular weights ranging from 25–71 kDa. Other common features for S-layers often found in *Bacillus* strains and its relatives are the S-layer homologous domains (SLH domains) that are located either in the N-terminal or in the C-terminal region and are involved in the anchoring of the S-layer to the cell wall components [22,23]. However, such domains are not present in other bacteria such as *Lactobacillus*.

The present study is focused on a strain that could be classified as *Viridibacillus arvi* (*V*. *arvi*) JG-B58 which was isolated from the Haberland uranium mining waste pile in 1997. *V*. *arvi* was firstly described by Heyrman, J. et al. (2005) [24] and is renamed from *Bacillus arvi* after new phylogenetic classification [25]. They isolated this strain from a Drentse A agricultural research area in the Netherlands. Albert, R.A. et al. (2007) isolated three strains of *V*. *arvi* from organic-matter-rich samples from a sphagnum bog, river sediments and forest soil, respectively, in south-eastern Wisconsin [25]. The cells are described as straight, round-ended and Gram-variable with motile rods and a size of 0.8–1.0 x 3.0–8.0 μm [24].

In a previous study, the cell surface of living cells of *V*. *arvi* JG-B58 was investigated by AFM analyses by Günther, T. J. and Suhr, M. et al. (2014) and the presence of S-layer proteins with square lattice structure was proven [26]. In the present work more detailed AFM analyses of the cell surface of aged cells of *V*. *arvi* JG-B58 discovered the presence of two different S-layer proteins. Furthermore, this study describes for the first time the *in-vivo* imaging of two superimposed S-layer proteins via AFM analyses. Sodium dodecyl sulfate polyacrylamide gel electrophoresis (SDS-PAGE) analyses of isolated proteins indicated the presence of three putative S-layer proteins of different molecular weights. More detailed genetic studies predict the presence of at least three different S-layer-like genes. All proteins show unique features in comparison to other S-layer proteins. The results assume that *V*. *arvi* JG-B58 expresses a novel type of S-layer proteins.

## Materials and Methods

### Bacterial strain and growth conditions

The rod-shaped strain JG-B58 (later classified as *V*. *arvi* JG-B58) was isolated from the uranium mining waste pile Haberland near Johanngeorgenstadt (Saxony, Germany) in 1997 [27,28]. The strain is held in our lab at Helmholtz-Zentrum Dresden-Rossendorf and was recovered from a cryo-preserved culture. All experiments were done under sterile conditions. The bacteria were cultivated under aerobic airflow conditions (dO_2_ level ≥ 30%) in a nutrient broth media (4 g/L meat extract, 4 g/L peptone, 2 g/L NaCl) at pH = 7.0 and 30°C in a 5 L scaled bioreactor (7 L System ADI 1030, Applikon Biotechnology B.V., Delft, Netherlands). *Online* parameters *e*. *g*. dissolved oxygen level (dO_2_), pH value, temperature and acid uptake rate were recorded by the software BioXpert V2 (Applikon Biotechnology B.V., Delft, Netherlands). Furthermore the bacterial growth was followed *online* by turbidity measurements (BugEye 2100, BugLab Concord, CA, USA). *Offline* optical density (OD) was determined by photometric measurements of the adsorption at 600 nm. Samples were measured against sterile filtrated NB-medium as blank values and after reaching adsorption >0.4 the cell suspension was diluted following the linearity of the Lambert-Beer law.

The progress of cultivation was controlled by taking cell samples directly from the bioreactor or the shaking flasks. *V*. *arvi* JG-B58 cells were obtained by centrifugation (9,000 x g, 4°C, 10 minutes) and were washed twice with a sterile-filtered phosphate buffered saline solution (PBS; 137 mM NaCl, 2.68 mM KCl, 8.1 mM Na_2_HPO_4_, 1.47 mM KH_2_PO_4_, pH = 7.4). The cells were suspended in PBS and the samples were split for microscopic studies, measurements of *offline* optical density and RNA analyses. Partially, the samples were shock frozen with liquid nitrogen and stored at -80°C up to the measurements. The *offline* optical density was measured photometrically at the adsorption maximum of *V*. *arvi* JG-B58 cells at 600 nm (Ultrospec® 1000, Amersham Pharmacia Biotech, Great Britain). Protein concentrations were measured at a wavelength of 280 nm and DNA absorbance at 260 nm (NanoDrop 2000/2000c UV/Vis Spectrophotometer, Thermo Fisher Scientific, USA). Phase contrast microscopy was done in 400 and 1000 fold magnification (BX61 Motorized research microscope, Olympus Germany LLC, Germany). The images were taken with the Cell^P imaging program (version 3.1, Olympus Soft Imaging Solutions LLC, Münster, Germany).

### S-layer protein isolation

Exponential growth phase cells of *V*. *arvi* JG-B58 were used for protein isolation. Two different methods were used to isolate the S-layer proteins. First, the S-layer proteins were isolated described in detail by Suhr, M. et al. (2014, 2016) and named as “standard” isolation method [29,30]. All steps were performed at 4°C.

The second isolation was done with an adapted and improved method of Lortal, S. et al. (1992) by using lithium chloride, that should lead to a selective and high isolation efficiency of S-layer proteins from the cell surface [31]. The cells of *V*. *arvi* JG-B58 were harvested by centrifugation (9,000 x g, 4°C, 20 minutes) and the pellet was washed twice with standard buffer. To 110–125 mg of the washed moist cell pellet 1 mL of cooled 5 M LiCl was added. The cell suspension was stirred for 1 to 1.5 h under permanent cooling at 0–4°C. The suspension was centrifuged for at least 60 minutes at 9,000 x g, and 4°C. The supernatant was dialyzed (MWCO 50,000) against ultrapure water at 4°C for at least 48 h with several changes of the dialysis liquid. After this, the content of the dialysis tubes were centrifuged at 47,500 x g, 4°C for at least 90 minutes. The white precipitate of S-layer proteins was dissolved in less ultrapure water and freeze dried.

### Protein characterization

SDS PAGE as described by Laemmli, U. K. (1974) [32] was used as purity control of the isolated S-layer proteins. Therefore, purified and freeze dried S-layer proteins of *V*. *arvi* JG-B58 were dissolved in ultrapure water and a urea stock solution was added to give a final concentration of 4 M urea. For SDS PAGE 10% separation gels were prepared and protein samples with a maximum amount of 10 μg proteins were injected in the pockets. The gels were stained by colloidal Coomassie Brilliant Blue G250 staining [33,34,35] and analyzed with the VersaDoc visualization system (Molecular Imager® VersaDoc MP 4000 System, Bio-Rad Laboratories, Inc.) with the program PD Quest.

Putative S-layer proteins were characterized with N-terminal protein sequencing. *V*. *arvi* JG-B58 S-layer proteins were separated in a 7.5% sodium dodecyl sulfate polyacrylamide gel and transferred to a PVDF membrane by the Western blot method [36,37] using the Trans-Blot Semi-Dry Electrophoretic Transfer cell (Bio-Rad). The blotted PVDF membrane was stained in Coomassie staining solution containing 0.1% of Coomassie R-250, 40% of methanol and 10% of acetic acid for 10 minutes. Afterwards the membrane was decolorized in a solution of 40% methanol and 10% acetic acid. The membrane was dried and the remaining protein bands of interest were cut and analyzed using an ABI 494A Procise HT sequencer (Applied Biosystems) at the Helmholtz Zentrum für Infektionsforschung Braunschweig.

### DNA and RNA analyses

The DNA of 1 ml overnight bacterial cell culture of *V*. *arvi* JG-B58 was isolated and purified using the MasterPure Gram-positive DNA Purification Kit (Epicentre) according to the manufacturer’s instructions. Purity and concentration of the DNA were determined using the NanoDrop system and agarose gel electrophoresis [38]. PCR amplifications were carried out using a T3 Thermocycler (Biometra). The synthesis of oligonucleotides was done by Eurofins MWG Operon. The sequences of all used oligonucleotides are listed in Table 1. All amplification reactions were performed in a volume of 20 μl using *Pfu* DNA polymerase (Fermentas) and the conditions were optimized for each primer pair. The obtained PCR products were purified using the Quickstep 2 PCR Purification Kit (Edge Bio Systems) and sequenced from each strand using a Perkin Elmer Applied Biosystems 377 instrument.

**Table 1:** PCR oligonucleotides.

gene	name	sequence 5’-3’	PCR product size
**JG-B58_*slp1***	B58_Slp1_45f	AGCAATCGGTGCAGCAGTAT	1662 bp
	B58_Slp1_1706r	GCTGAATCGCCGTTAGCATC	
**JG-B58_*slp2***	B58_Slp2_744f	AAACGGTGTTGAAGTTGGCG	1696 bp
	B58_Slp2_2439r	TGCAGTAGCAACATCACCTGT	
**JG-B58_*slp2***	B58__Slp3_259f	CCGACTGGGATATACGCTAGG	1055 bp
	B58_Slp3_1313r	GCCTCCACCTTTTGGATCGTA	
***16S***	7f	AAGAGTTTGATCNTGGCTCAG	1500 bp
	1513r	TACGGYTACCTTGTTACGACTT	

The sequencing of the whole genome of *Viridibacillus arvi* JG-B58 was performed using the Next Generation Sequencing (NGS) technology with the Illumina Hi Seq 2000 by AROS Applied Biotechnology A/S. The used Illumina Hi-Seq 2000 technology provided read lengths of 2 x 100 base pairs (bp) for the whole genome within a run time of 8 days. Bioinformatic analyses were performed with the Genomics Workbench (CLC bio) as previously described [39].

Total RNA of *V*. *arvi* JG-B58 was isolated from a bacterial culture after 2.5, 3, 4, 5, 6, 7, 8, 9, 10 and 25.5 h of growth. Ten millilitres of the bacterial suspension were harvested by centrifugation. The cell pellet was resuspended in 100 μl TE buffer containing 10 mM Tris-HCl and 1 mM EDTA at pH = 8.0. After addition of 6 μl Lysozym (50 mg ml^-1^) the Gram-positive bacteria were incubated at 30°C for 30 minutes in order to pre-lyse the cells. Afterwards the total RNA-isolation was performed using the innuPREP RNA Mini Kit (Analytik Jena). Residual DNA was digested using the innuPREP DNaseI Digest Kit (Analytik Jena). The isolated RNA was dissolved in 30 μl RNase free water. The OD_260_ value was measured spectrophotometrically with the NanoDrop 2000/2000c UV/Vis Spectrophotometer (Thermo Scientific) in order to determine the total RNA concentration and purity. Additionally, the success of total RNA purification was controlled with agarose gel electrophoresis. The reverse transcription of mRNA to cDNA was performed using the Maxima Reverse Transcriptase (Thermo Scientific). The resulting cDNA samples were placed on ice until their usage in PCR reactions with the oligonucleotides shown in Table 1. As positive control cDNA was amplified with 16S primers. As negative control in order to check DNA contaminations, RNA was used as template and incubated with 16S primers (Table 1). As another positive control PCR was performed using S-layer specific primers and genomic *V*. *arvi* JG-B58 DNA as nucleic acid template.

All gene sequences reported in this paper have been deposited in the EMBL database. The EMBL accession numbers are LN867316 (*V*. *arvi slp*1), LN867317 (*V*. *arvi slp*2), and LN867318 (*V*. *arvi slp*3).

### Atomic force microscopy

For S-layer recrystallization 5 x 5 mm pieces of a silicon dioxide wafer (AMD Saxony LLC & Co. KG, Dresden, Germany) were used and cleaned by using the RCA method [40]. After this the SiO_2_ surfaces were modified by the layer by layer (LbL) technique [41,42] using polyelectrolytes as adhesion promoter to obtain a homogenous and stable surface charge. The preparation of living cells was done as previously described by Günther, T. J. and Suhr M. et al. (2014) [26]. The coating with isolated S-layer proteins was done by a method previously described [26,29,30]. Freeze dried S-layer proteins of *V*. *arvi* JG-B58 were dissolved in ultrapure water and a urea stock solution was added to a final concentration of 4 M urea. For recrystallization the proteins were transferred to the recrystallization buffer in a final concentration of 0.2 g/L and incubated for at least 1 h at room temperature.

AFM images were taken with an MFP3D Bio (Asylum Research, Santa Barbara CA) using the AC mode in liquid (PBS for cells and ultrapure water for recrystallized S-layer proteins). The Cantilever (Biolever mini BL-AC40TS-C2, Olympus Life Sciences) in AC mode was used for all measurements. Therefore a closed fluid cell (BioHeaterTM, Asylum Research, Santa Barbara CA) with a total volume of 1.5 ml was used. The temperature of the cell content was kept constant at 25°C and scanning speed was adjusted between 2.5 and 10 μm/s.

## Results

### Bacterial strain characterization and cultivation

The strain *Viridibacillus arvi* JG-B58 that was previously isolated from soil samples [43] was characterized by 16S rDNA analyses. The partial 16S rRNA sequences showed 100% identity to *V*. *arvi* strain LMG 22165 (accession no. AJ627211) [24,25] isolated from soil sample and to *V*. *arvi* strain 433-D9 (former classified as *Bacillus psychroviridis* strain 433-D9; accession no. AY266991) [25]. Phase contrast microscopic analyses reveal that the cells of *V*. *arvi* JG-B58 are straight, round-ended and have a size of 0.8–1.0 x 3.0–5.0 μm, thus showing similarities to *V*. *arvi* cells as described by Heyrman, J. et al. (2005) [24] and Albert, R. A. et al. (2007) [25].

**Fig 1:**
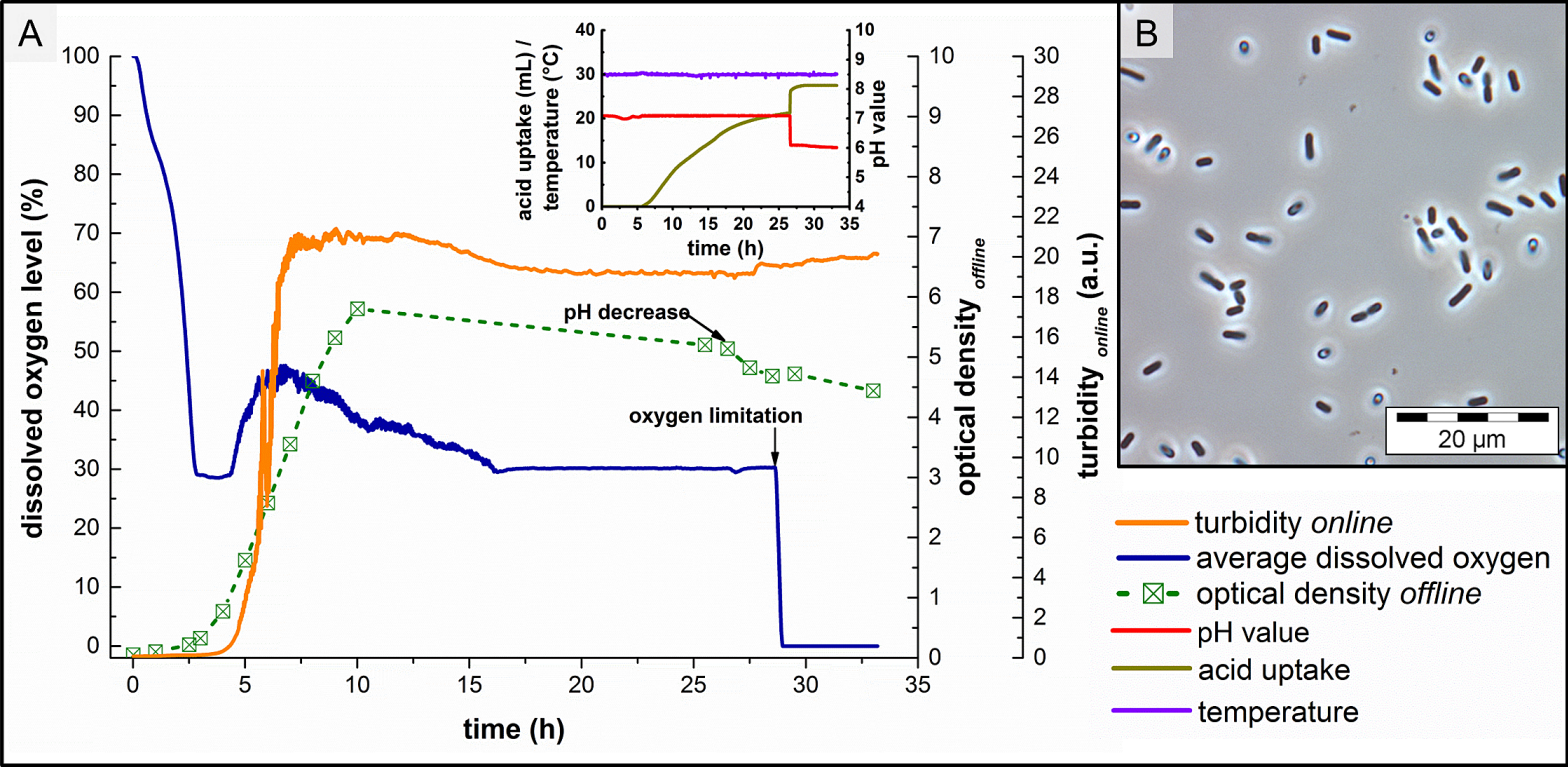
Cultivation of *V*. *arvi* JG-B58. (A) *Online* and *offline* cultivation parameters including changes in pH level and oxygen availability for bacterial stress response and (B) microscopic image of undiluted (OD_600 nm_ = 2.5) exponential growth phase cells (5 h) of *V*. *arvi* JG-B58 in 1000 fold magnification.

The expression of S-layer proteins severely depends on different parameters like growth stage and nutrition conditions [44]. Therefore, cultivation of *V*. *arvi* JG-B58 and S-layer synthesis was monitored in detail. Based on these analyses, optimal growth conditions and the optimal harvesting time were estimated. As shown in Fig 1A the cells of *V*. *arvi* JG-B58 enter the exponential phase after ≈3 h lag phase, where the growth rate of cells reached its maximum. An exemplary microscopic image of vital not sporulated cells at 5.5 h is shown in Fig 1B.

After 5.5 h the uptake of acid marks the transition to the retarding phase of bacterial growth. Therefore, half of the cells were harvested under sterile conditions after approximately 5.5 h and biomass was used for S-layer protein isolation to obtain the maximum amount of S-layer proteins.

For the investigation of the expression of the different S-layer proteins at the different growth phases, the cultivation and sampling was continued up to a total time of 35 h. After ≈7.5 h the cells entered the stationary growth phase detected by almost constant signals of the *online* turbidity and decreasing signals of the dO_2_ level. The small decrease in *online* turbidity and *offline* measured OD after 10 h of cultivation are an indicator for the beginning starvation of *V*. *arvi* JG-B58 cells.

### S-layer protein isolation

The presence of S-layers was investigated on molecular level. For these investigations the protein lattices were isolated using the”standard” procedure and LiCl method. Both methods were compared and evaluated regarding protein yield, protein purity and quality. Further, the influence on recrystallization behavior on technical surfaces depending on preparation method was tested.

**Fig 2:**
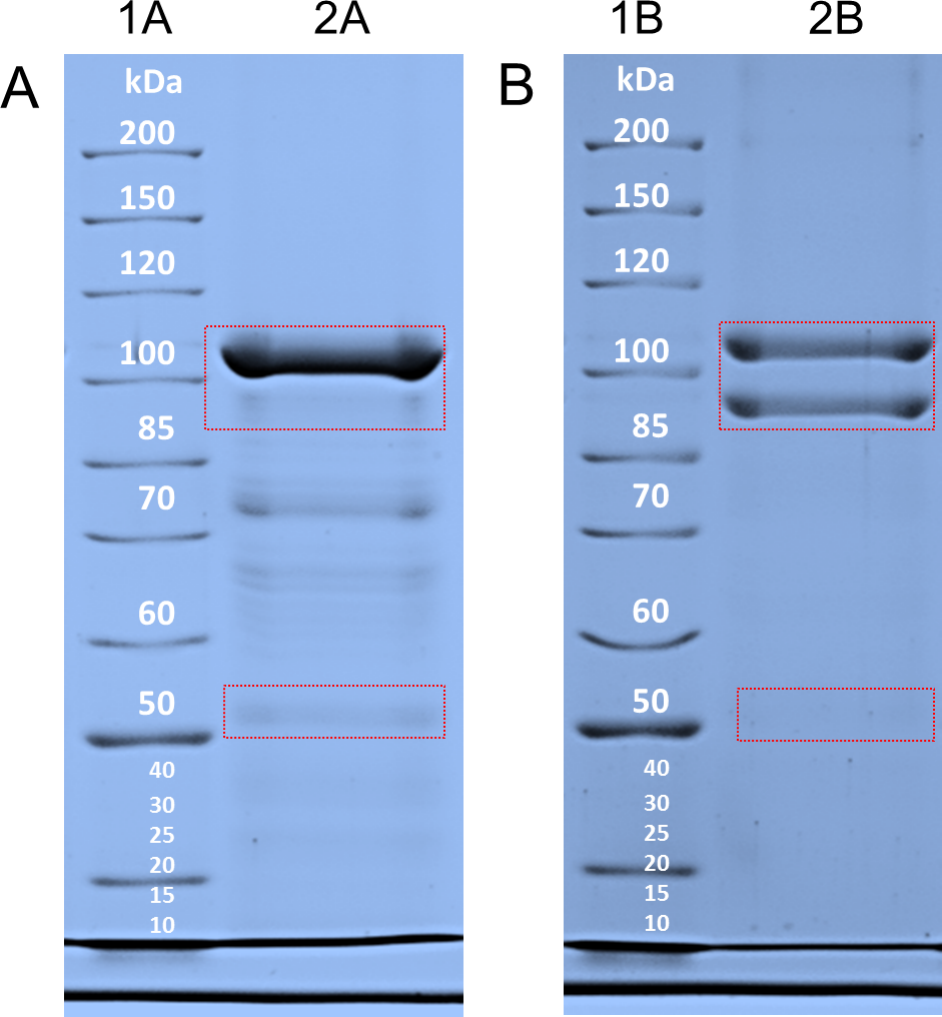
SDS-PAGE pattern of isolated S-layer proteins of *V*. *arvi* JG-B58. (A) SDS gel; lane 1A: molecular mass standard, lane 2A: 10 μg protein (“standard” isolation), (B) SDS gel lane; 1B: molecular mass standard, lane 2B: 10 μg protein (LiCl isolation)–marked region around 100 kDa for Slp1 and Slp2 and at 55 kDa for Slp3.

For the experiments, exponential growth phase cells (after approximately 7 h cultivation, see Fig 1) were harvested and underwent both methods of S-layer protein isolation. SDS-PAGE was performed in order to control the purification process as well as to analyze the molecular weights of purified S-layer proteins obtained by the two methods (Fig 2).

In Fig 2A, showing protein patterns of S-layer preparations using the “standard” method, one major protein band is visible at a molecular weight of about 105 kDa. A second weak band appears with a size of about 53 kDa being probably also an S-layer protein (for this see paragraph “gene analyses”). Further, some impurities can be seen. In Fig 2B, showing protein patterns of S-layer preparations using the LiCl method, two major bands corresponding to molecular weights of about 105 kDa and 95 kDa are visible. Furthermore, an additional weaker signal was detected with a molecular weight of about 53 kDa. Other bands were not visible, indicating that this purification method allowed the preparation of S-layer proteins with high purity. The three proteins were assigned as Slp1 (105 kDa), Slp2 (95 kDa), and Slp3 (53 kDa).

### Recrystallization of S-layer proteins on surfaces and AFM studies

The proteins that were isolated by both isolation methods were further characterized by AFM analyses in order to verify their S-layer nature. Such studies are suitable to give information on protein structure, self-assembling properties and lattice symmetry that is not accessible by the prior used methods. In addition to the analyses of isolated proteins, the surfaces of living cells were visualized by AFM.

Generally, S-layer proteins possess the ability to intrinsically self-assemble spontaneously on technical surfaces, thereby forming a two dimensional protein lattice on surfaces, in suspension or at interfaces [45]. Previous studies proved that this spontaneous self-assemblage can be significantly improved by a prior modification of the surfaces with polyelectrolytes [26,29,30]. Therefore in the present work we used the same method for *in vitro* assembly of *V*. *arvi* JG-B58 S-layer as described for SlfB, the functional S-layer protein of *L*. *sphaericus* JG-A12, and for Slp1 of *L*. *sphaericus* JG-B53 [29,30,46]. A mixture of proteins of Slp1, Slp2, Slp3 (see previous chapter), obtained by the two different isolation methods was adjusted to 0.2 mg protein/ml and 1.0 mM calcium in recrystallization buffer. From both samples AFM images were taken and the recrystallization of the proteins obtained by both isolation methods was compared.

**Fig 3:**
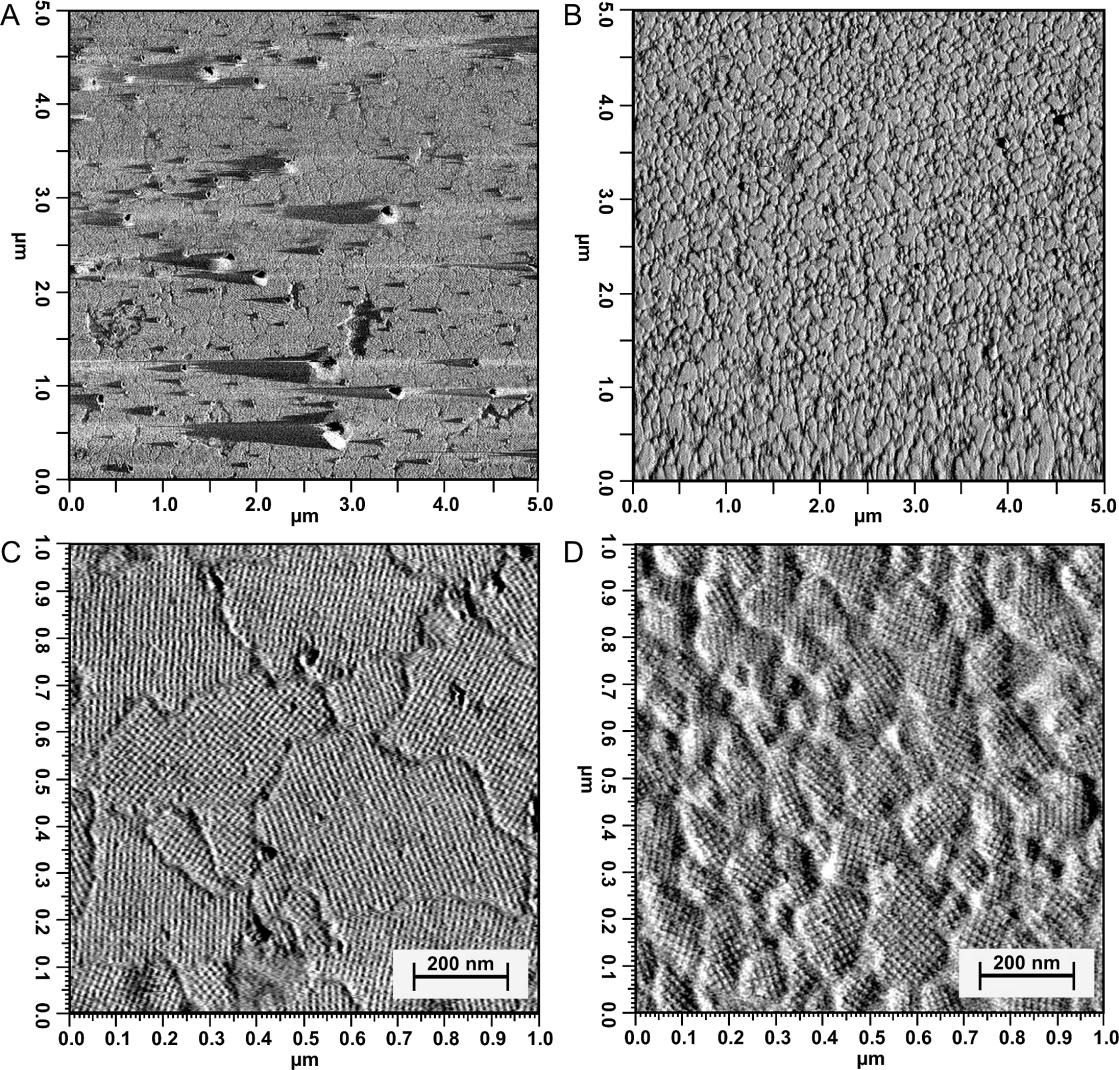
AFM images of recrystallized *V*. *arvi* JG-B58 S-layer proteins. AFM amplitude images (5x5μm) of recrystallized S-layer proteins (A) isolated using adapted “standard” method and (B) using chaotropic salt (LiCl); detailed AFM amplitude images (1x1 μm) of recrystallized S-layer proteins (C) isolated using adapted “standard” method and (D) using chaotropic salt (LiCl).

Fig 3A and 3C show amplitude images of self-assemblies of S-layers obtained by the “standard” isolation method. The surface of the substrate is almost completely covered with the S-layer patches with a thickness of 8 nm and a lattice constant of 14.2 nm ± 0.4. These patches are relatively large, forming arrays with squared (*p4*) symmetry comparable to the surface of the bacterial cells (Chapter Visualization of vital bacterial surfaces by AFM). From SDS-PAGE results it can be suggested that these arrays are mainly composed of the protein Slp1 (see previous chapter). In Fig 3A, additional protein agglomerates are visible that are attached to the S-layer arrays. These agglomerates are possibly either misfolded S-layer proteins or impurities remaining from isolation that are also visible in the protein profile (Fig 2A).

Fig 3B and 3D show amplitude images of S-layers obtained by the method using the chaotropic salt lithium chloride. Also in this case the surface of the substrates is completely covered with the protein arrays. Remarkably, the S-layer patches are significantly smaller than in Fig 3A and 3C, also forming arrays with square symmetry and a lattice constant of 16.3 nm ± 0.8, thus slightly larger than in Fig 3A and 3C. In addition, protein agglomerates that are attached to the surface are not visible, confirming that this isolation procedure results in less impurity that disturbs the coating process. The protein solution contains the different putative S-layer proteins Slp1, Slp2, and Slp3. Therefore, a combined and simultaneously recrystallization of Slp1, Slp2 and Slp3 cannot be excluded, as well as the possibility that only one of the found proteins is able to spontaneously self-assemble on surfaces. More detailed analyses of self-assembly properties of each protein and verification of postulated symmetries require the isolation of the respective protein followed by recrystallization and AFM analyses.

### Visualization of vital bacterial surface by AFM

**Fig 4:**
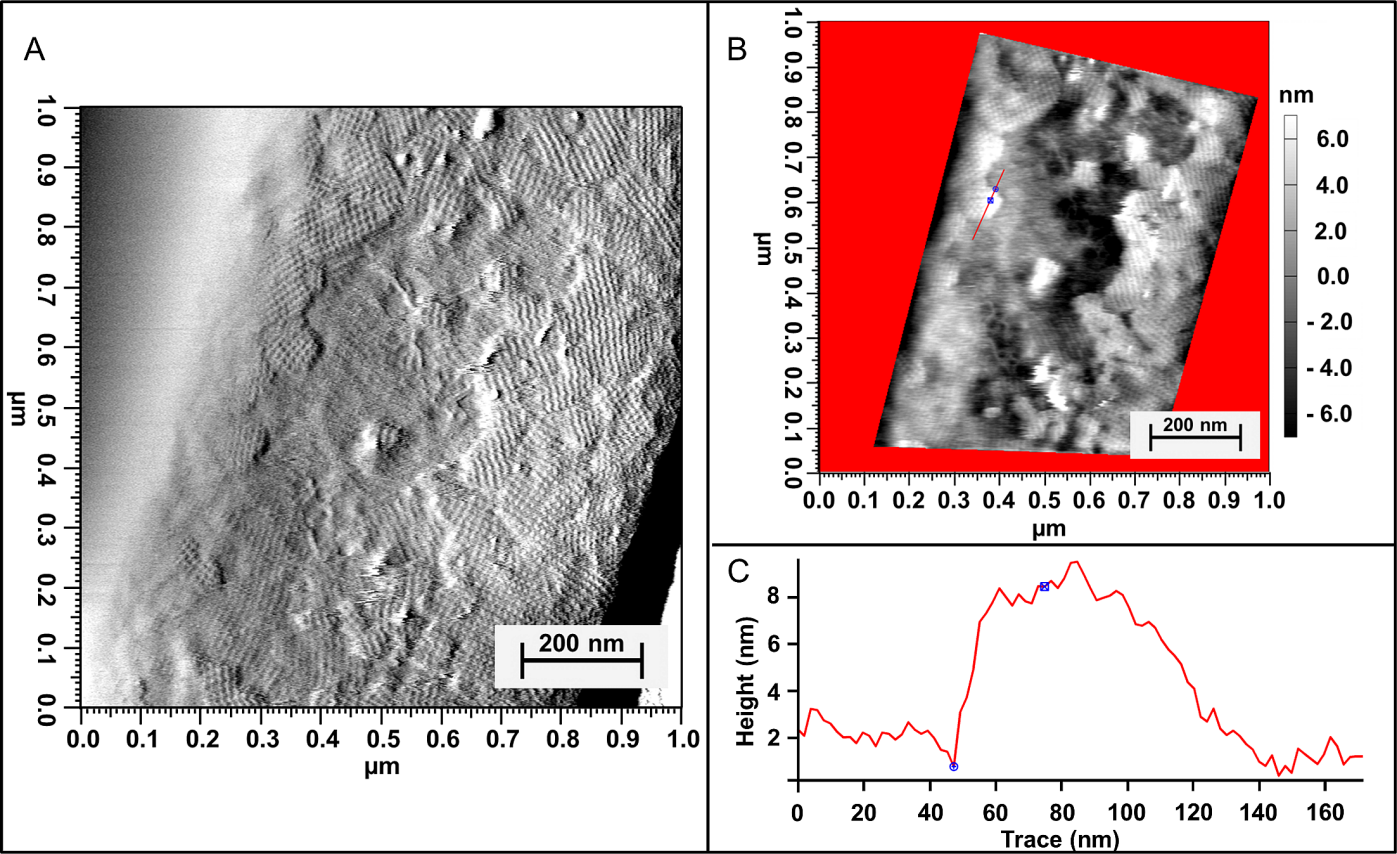
AFM images of living cells of *V*. *arvi* JG-B58. Bacterial cells are immobilized on polyelectrolyte modified SiO_2_ substrates performed by the method described by Günther, T. J. and Suhr, M. et al. (2014) [26]; (A) amplitude image of an aged cell showing partial S-layer protein detachment, (B) AFM height image of the sample; the bar marks the area for surface profile analyses (C) surface thickness profile of the region showing partial S-layer protein detachment.

AFM was used for cell imaging and high resolution imaging of surface properties of living bacterial cells under physiological conditions. For such analyses we have previously implemented a new sample preparation method that allows the *in vivo* AFM imaging of the cell surface of a wide range of different microorganisms [26]. In simplified terms, the immobilization of living cells of *V*. *arvi* JG-B58 was realized by centrifugation of a diluted cell suspension on polyelectrolyte coated surfaces. The used method has been proven to be a reliable and efficient way not only for the immobilization of microorganisms but also for the imaging of the S-layers on living cells of *L*. *sphaericus JG-B53* and *V*. *arvi* JG-B58. Subsequent to the previous AFM investigation of *V*. *arvi* JG-B58 more detailed studies were done. Therefore, not only vital exponential growth phase cells were studied by AFM, but also aged cells under limitation of nutrients and oxygen were investigated. The images prove the presence of two proteinaceous layers at the surface of aged cells. The AFM images and surface profiles are shown in Fig 4.

In comparison to the AFM imaging of vital cells of *V*. *arvi* JG-B58 by Günther, T. J. and Suhr, M. et al. (2014) that shows uniformly closed S-layer protein layers [26] the aged cells that were investigated in the present study possess a disordered protein envelope. Rather, the amplitude image (Fig 4A) shows that the surface of the aged cells is composed of smaller crystallites and is not fully covered. Additionally, the amplitude image presents a partial peeling of the upper highly ordered surface layer lattice. The outermost protein layer possesses square symmetry (*p4*) with a lattice constant of 13.3 ± 0.3 nm. Below the upper striped layer a second protein grid with a well-defined orientation became visible. The orientation of this underlying second layer also possesses squared symmetry with a comparable lattice constant but does not follow the orientation of the upper layer. This leads to the conclusion that it is a fully standalone protein layer and not an artifact or imprint of the upper layer. AFM height image and surface thickness profile visualized in Fig 4B and 4C determined the thickness of the peeled upper proteinaceous surface layer with approximately 8 nm. This value corresponds to previous data obtained from studies for the determination of monolayer height of *V*. *arvi* JG-B58 isolated S-layer proteins (this study).

The presented AFM results prove that *V*. *arvi* JG-B58 exhibits a double S-layer. This is the first report proving the existence of a double S-layer on living cells.

### Gene analyses

Genome analyses were performed in order to identify all S-layer protein gene sequences encoded by the strain. The *de novo* sequencing of 0.2 mg dsDNA of *V*. *arvi* JG-B58 yielded 56.7 million purified filtered sequence reads, which were used to create 298 contigs and to identify its total genome size of 4.7 million base pairs. Putative ORF and deduced protein sequences were gained from analyses of genomic data with CLC Genomics workbench (CLC bio). The results of N-terminal sequencing of purified S-layer proteins were used for the identification of S-layer protein sequences. Three N-terminal sequences of dominant bands that were obtained at SDS-PAGEs after protein purification were determined as KAATVKVSKGKLVSAKSG, KAVKGYKSYKGVLY and KSSTSVKVSNGKLVY. The first two sequences could be assigned to the N-terminus and an internal region of an S-layer protein referred to as Slp1, the third sequence could be assigned to the N-terminus of another S-layer protein, referred to as Slp2. A fourth sequence of a weak band at the SDS-gel was determined as KVSNVL. This sequence could be assigned to an internal sequence motif of a putative S-layer protein referred to as Slp3. Sequence comparisons with known S-layer protein genes confirmed the suggested homology with S-layer protein genes. The sequences of all three genes are deposited in EMBL database (Acc. No. LN867316, LN867317, LN867318). General features and comparison with related S-layer proteins are summarized in Table 2. Slp1 and Slp2 have a similar theoretical molecular weight of 105.4 and 103.2 kDa, respectively, whereas Slp3 is much smaller with a theoretical molecular weight of 53.4 kDa. The amino acid composition of Slp1 and Slp2 shows typical S-layer features such as the absence of cysteines, a low content of histidine, tryptophane and methionine, and a high content of threonine. Remarkably, Slp3 possesses one cysteine.

**Table 2:** S-layer homologous proteins of *V*. *arvi* JG-B58 and comparison with other proteins from database.

S-layer	Similarity (BLAST)	Protein size (aa)	Molecular mass (kDa) (mp)	calculated pI (mp)	Signal peptide (aa)	SLH domains and location
***V*. *arvi* JG-B58 Slp1**	**---**	**1,016**	**105.4 (101.7)**	**9.29 (9.25)**	**37**	**none**
***B*. *isronensis*** (WP_008408804.1)	51 %	1,259	131.3 (128.2)	5.35 (5.19)	31	1 (N)
***L*. *fusiformis*** (WP_009371904.1)	37 %	1,139	119.7 (116.7)	5.1 (4.99)	30	3 (N)
***V*. *arvi* JG-B58 Slp2**	**---**	**983**	**103.2 (99.3)**	**8.75 (8.56)**	**39**	**none**
***V*. *arenosi* FSL R5-213]** (ETT87905)	53 %	984	103.3 (99.8)	9.19 (9.15)	36	none
***V*. *arvi* JG-B58 Slp3**	**---**	**463**	**53.4 (49.4)**	**8.97 (7.84)**	**35**	**none**
***L*. *sphaericus* JG-B53 Slp2** (AGG82437.1) [39]	89 %	728	82.9 (78.9)	6.13 (5.72)	35	1 (C)
***Bacillus mycoides* DSM 2048** S-layer domain protein (EEL96495.1) [47]	79% (Pos. 22–461)	728	82,5	6,32	none	N.D.

aa: amino acid; kDa: kilo Dalton; mp: mature protein; pI: isoelectric point; SLH domain: S-layer homologous domain; N: N-terminal; C: C-terminal

Despite these typical features, all three S-layer proteins have some other characteristics that differ from S-layer proteins of the *Bacillus* type. They possess higher calculated pIs ranging from 8.75 to 9.29 and SLH domains that are responsible for anchoring to the cell wall are missing. All three putative S-layer proteins of JG-B58 have signal peptides of 35–39 aa that are responsible for the secretion to the cell surface. The theoretical molecular masses of the mature proteins are 101.7 kDa for Slp1, 99.3 kDa for Slp2, and 49.4 kDa for Slp3. These sizes are approximately in accordance with the masses determined by SDS gel electrophoresis.

**Fig 5:**
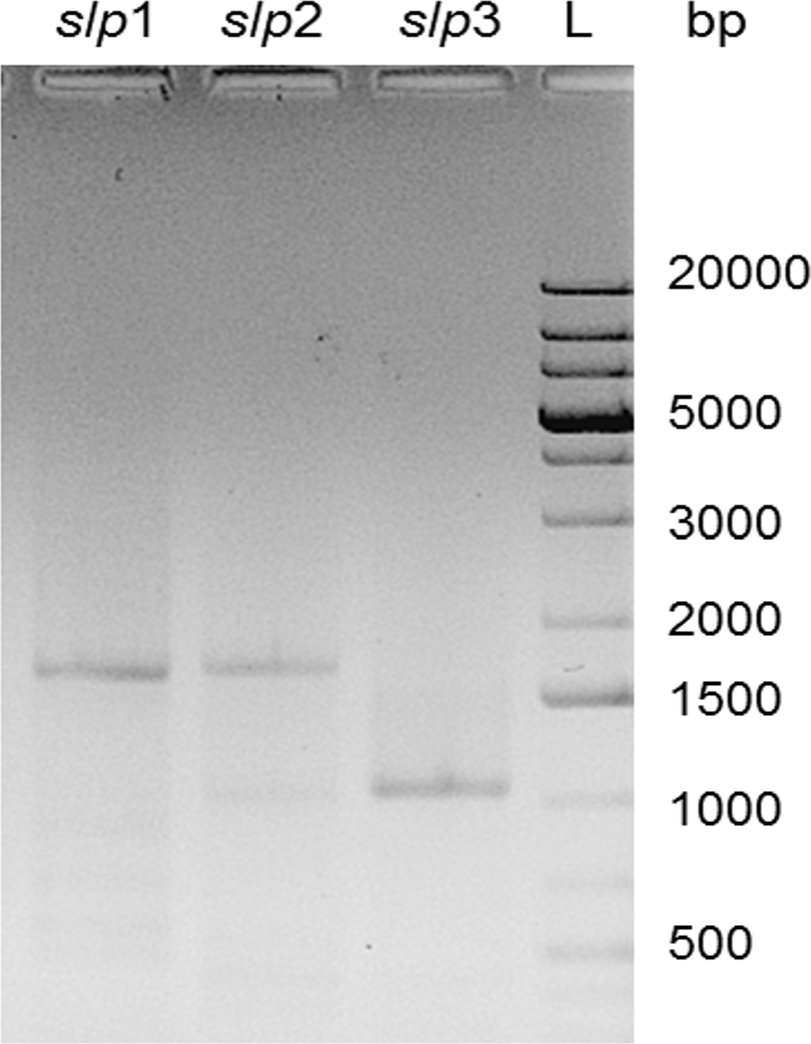
Gene expression. Analyses of PCR products using cDNA of *V*. *arvi* JG-B58 cells after 25.5 hours of growth as template. Lanes *slp*1 (1662 bp), *slp*2 (1696 bp), *slp*3 (1055 bp). Lane L, GeneRuler 1 kb Plus DNA Ladder (Fermentas).

Analyses with the basic local alignment search tool (BLAST) of Slp1 showed high similarities to other predicted S-layer proteins *e*. *g*. from *Bacillus isronensis* (51% identity) and *Lysinibacillus fusiformis* (37% identity). However, these proteins are with molecular weights of 131.3 and 119.7 kDa larger then Slp1, have lower “S-layer-typical” pIs of 5.35 and 5.1 and possess one or three SLH-domains, respectively. BLAST analyses of Slp2 showed a high similarity (53% identity) with a hypothetical S-layer protein of *Viridibacillus arenosi* FSL R5-213, the same genus as described in this study. This protein has similar features as Slp2: a relatively high pI of 9.19, a similar size and the absence of SLH-domains. However, the sequence is just given in the database, an expression or further investigation of the protein is not described. BLAST analyses of Slp3 showed a high similarity (89% identity) with the first 462 amino acids of the putative S-layer protein Slp2 of *L*. *sphaericus* JG-B53, an S-layer protein that has been determined in a previous genome analysis in our lab by Lederer, F. L. et al. (2013) [39]. The complete Slp2 of JG-B53 possesses a significant lower calculated pI of 6.13 than the protein of JG-B58 and a C-terminal SLH domain has been identified. In *L*. *sphaericus* JG-B53 the protein was not expressed during cultivation. Further, Slp3 showed a high similarity with different postulated S-layer proteins of members of the *B*. *cereus* group (e.g. *B*. *mycoides* DSM 2048 [47], 79% identities of aa 22–461) (Table 2). It has to be mentioned, that an expression of all these proteins that were found in the database has not been confirmed yet and information on self-assembling properties is missing. Therefore the meaning of the additional C-terminal amino acids for protein structure and functionality is unknown. The upstream regions of all three genes of *V*. *arvi* JG-B58 comprise typical ribosomal binding sites and two putative promoter sequences, indicating that all proteins are functional. Analyses of mRNA were performed in order to investigate the expression of the genes during different growth stages. The presence of PCR products from cDNA in all three cases demonstrated that principally all three genes are expressed during bacterial growth, at late growth stage with almost the same expression level (Fig 5).

## Discussion

### Isolation of S-layer proteins using two different approaches

In the present study two different procedures for S-layer isolation were applied. The first method, called “standard” procedure, was based on cell disruption followed by subsequent extraction and centrifugation steps. In the past this method was shown to facilitate a very effective S-layer isolation giving a high protein yield. The second method used the chaotrophic salt LiCl for dissolution of S-layer proteins of the cell wall without disruption.

Comparison of both isolation procedures suggests the assumption that the use of chaotrophic salts is a mild method allowing the removal of surface proteins from the cell wall without causing protein contaminations. In contrast, total cell disruption as performed in the first method allows the isolation of large amounts of S-layer-proteins, but release of cytosolic proteins causes impurities. Interestingly, the S-layer pattern differs between both isolation methods. It seems that the “standard” procedure is not suited for the purification of Slp2 and Slp3. It can be assumed that they are associated with other cell proteins or stay in the supernatant and are not part of the separate white S-layer containing layer that is visible during the isolation procedure. In Fig 2B, band intensities of Slp1 and Slp2 suggest a similar expression whereas the weak band of Slp3 indicates a low amount. This is contrary to the cDNA analyses that indicate a similar expression level of all three proteins. One can suggest that Slp3 is possibly anchored more strongly to underlying cell wall components *e*. *g*. the peptidoglycan and cannot be isolated by the lithium chloride method or gets lost during dialysis. However, whether these differences can be attributed to the preparation methods has to be investigated in future. In conclusion, the protein patterns indicate the presence of three S-layer proteins of different sizes. Further, the LiCl method seems to be the more appropriate method for S-layer preparation in case of *V*. *arvi* JG-B58.

### Recrystallisation experiments

It is well known that the self-assembly process of S-layer proteins is influenced by protein concentration, but also by different bivalent cations such as Ca^2+^ and Mg^2+^ [48,49,50,51]. Furthermore, also the properties of the surface of the substrates influence protein adsorption. Literature typically describes S-layer proteins with a negatively-charged inner surface with a rough topology and an outer surface that is almost smooth and uncharged [46]. Thus, polarity and the density of surface charges on the targeted S-layer influence coating kinetics and the covering ratio during the recrystallization process. Our experiments with *V*. *arvi* JG-B58 S-layer mixture confirmed previous statements that a positively charged polyelectrolyte layer as closing layer improves the S-layer protein coating [26,29,30]. Proteins obtained from both isolation methods completely covered the thus modified SiO_2_ surface. Furthermore in comparison to other bacteria (e.g. *L*. *sphaericus* JG-A12) the S-layer proteins of *V*. *arvi* JG-B58 exhibit excellent recrystallization characteristics (fast and reproducible), coating kinetics and layer stabilities at such surfaces. One can speculate that the presence of bivalent cations like Ca^2+^ stabilizes the self-assemblies of *V*. *arvi* JG-B58 S-layer proteins resulting in large patches (Fig 3A and 3C) as described for SbpA of *L*. *sphaericus* CCM2177 S-layer [52,53,54] whereas the presence of monovalent Li^+^ may disturb the coherent protein lattice assembly resulting in smaller patches (Fig 3B and 3D).

### Analyses of S-layer expression

*In-vivo* AFM analyses of cells of *V*. *arvi* JG-B58 revealed the presence of at least two major outer protein layers. More detailed analyses on protein and gene level proved the expression of the three putative S-layer proteins Slp1, Slp2, and Slp3 with distinct primary structures, molecular masses and pI values.

Further, the purified S-layers were recrystallized on SiO_2_ wafers and structural analyses showed that they exhibit arrays with *p4* symmetry. However, these analyses could not clearly resolve which of the three S-layer proteins is responsible for the respective layer. Further, analyses of the self-assemblies of each isolated protein have yet failed. From sequence comparisons and protein data one can speculate that at least both Slp1 and Slp2 (the dominant proteins) self-assemble to S-layer arrays with *p4* symmetry. However, further investigations are necessary.

There are several publications reporting the presence of double S-layers. Yamada, H. et al. (1981) reported the presence of two major outer protein layers in *Brevibacillus brevis* 47 during all growth stages [19]. Two superimposed, structurally different S-layers were detected for *B*. *brevis* strains CCM 1089 and CCM 1463 with molecular weights of 102 and 118/119 kDa [20]. In both organisms the outer S-layer lattice showed oblique and the inner S-layer lattice hexagonal symmetry. Similarly, also *Aquaspirillum serpens* and *Brevibacillus brevis* 41 exhibit two S-layer proteins [19,55]. Cerquetti, M. et al. (2000) monitored the presence of two superimposed structurally different S-layer lattices of hexagonal and square symmetry in different isolates of the anaerobic gram-positive bacterium *Clostridium difficile* [17]. From each strain two distinct glycosylated S-layer proteins could be isolated ranging in molecular mass from 35 to 56 kDa. Both proteins in a protein extract self-assembled to sheet exhibiting hexagonal symmetry after removal of the dissociating agent. Interestingly, HPLC purified proteins lost their self-assembling capability. However, in all these cases molecular details of the proteins were not given and self-assembly studies of isolated proteins were not performed.

Principally numerous functions have been assigned to S-layer proteins, *e*. *g*. protecting the cells from environmental stress, stabilizing the cells etc. Keeping in mind that the production of at least two different S-layers means an enormous consumption of energy, both S-layers should have a high relevance for the cells and fulfill essential functions. However, the specific functions and meaning for the cells require more investigation. It is known that several bacteria can produce an excess of S-layer proteins to ensure the complete coverage of the cell wall during all growth phases, and or either store excess of S-layer proteins in the peptidoglycan layer or secrete it into the environment. This full coverage of bacteria surface with S-layer proteins during the complete growth cycle could be visualized by electron-microscopic analyses of the cells [8]. In some strains, expression of S-layer variants depends on environmental conditions. For example, the *Geobacillus stearothermophilus* PV72 usually expresses a hexagonal S-layer protein. Oxygen stress induces the expression of another protein variant with *p2* symmetry, and the *p6* lattice is converted to a *p2* lattice [56]. In case of *V*. *arvi* JG-B58, the expression of all three putative S-layer proteins seems to be relatively independent from cultivation conditions. Variation of expression level may occur, but this has to be analyzed in detail.

In summary, it could be shown for the first time that exponential growth phase cells of *V*. *arvi* JG-B58 are able to express three different S-layer like proteins. Furthermore, SDS-PAGE pointed out differences between the two used protein isolations. Further attempts to separate the single proteins according to their different molecular weights/sizes by fast protein liquid chromatography (FPLC) or differences in their pI by two-dimensional gel electrophoresis has not been successful up to now. Therefore, a mixture of isolated Slp1, Slp2 and Slp3 were used for further recrystallization experiments. However, for a deeper understanding of the functions of the different S-layers as well as recrystallization properties and structural features the separation and individual analysis of each type of protein is crucial.

### Gene analyses

Genetic analyses confirmed the expression of three different S-layer-like protein genes. The corresponding gene products showed some unique features differing from other related bacterial strains raising new questions regarding the function of the S-layer proteins for the cells as well as evolutionary aspects. All three predicted S-layer proteins of *V*. *arvi* JG-B58 have a relatively high pI value ranging from 7.84 to 9.29 that is typical for *Lactobacillus*-S-layer proteins, ranging from 9.35–10.4 [21]. However, *Lactobacillus* S-layer proteins are smaller proteins with molecular weights ranging from 25–71 kDa. In contrast, the pI of *Bacillus*-type S-layer proteins ranges from 4 to 6 and the protein size is larger [3]. Only in few cases, e.g. in case of *Bacillus cereus*, a higher pI has been reported. For example, genome analyses of *Bacillus cereus* ATCC14579 [57] identified a putative S-layer of a size of 577 aa (Acc. No. AAP07978) that possesses three N-terminal SLH domains and exhibits a pI of 8.1 [58]. Another remarkable feature of Slp1, Slp2, and Slp3 is the absence of SLH-domains that recognize a distinct type of secondary cell wall polymers (SCWP), which carries pyruvic acid residues [3]. These domains can be typically found in S-layers of *Bacillus* relatives but lack in other species e.g. Lactobacilli. On the other hand, sequence comparisons show close relations to *Bacillus*-type S-layer proteins.

Horizontal gene transfer has been suggested for many S-layer protein genes. For example *L*. *sphaericus* JG-B53 exhibits at least 13 putative S-layer protein genes which show with one exception distinct similarities to S-layer protein genes of *Lysinibacillus* strains [39]. In *L*. *sphaericus* JG-A12 an intragenic patchwork evolution of the functional S-layer genes has been suggested [59]. Generally, horizontal gene transfer and rearrangements of gene segments have been postulated to be responsible for S-layer gene variations enabling the adaption to different stress factors [56,60] or contributing to their virulence [61,62,63,64].

It can be assumed that in case of *V*. *arvi* JG-B58 an intergenera horizontal gene exchange occurred followed by rearrangements of DNA segments resulting in a genetic patchwork with S-layers showing features of *Lactobacillus* and *Bacillus*-type proteins.

## Conclusion

*In vivo* AFM analyses of the cell surface of the bacterial isolate *Viridibacillus arvi* JG-B58 visualize the presence of two superimposed S-layers both exhibiting square symmetry. Protein and gene analyses prove the concomitant expression of the three different putative S-layer proteins Slp1, Slp2, and Slp3 with theoretical molecular weights of 102, 99, and 49 kDa. BLAST analyses show similarities to other S-layer proteins. More detailed gene analyses found that all three proteins exhibit typical S-layer signal sequences but do not contain SLH domains. Further, all proteins possess a relatively high pI value ranging from 7.84 to 9.29. In conclusion, *V*. *arvi* JG-B58 expresses a novel type of S-layer proteins showing features of *Lactobacillus* as well as of *Bacillus*-S-layer proteins. For S-layer preparation two different methods were applied and compared. The first “standard” procedure implied a disruption of the cells and removal of the S-layer proteins from the cell wall fragments. With this method high protein yields of Slp1 were obtained. In contrast, a mixture of all three proteins was obtained with the second LiCl-method. This method should be preferred for further future studies aimed at the separation of each protein type.
